# Lung clearance index to characterize clinical phenotypes of children and adolescents with cystic fibrosis

**DOI:** 10.1186/s12890-022-01903-5

**Published:** 2022-04-01

**Authors:** Simone Gambazza, Federico Ambrogi, Federica Carta, Laura Moroni, Maria Russo, Anna Brivio, Carla Colombo

**Affiliations:** 1 Department of Clinical Sciences and Community Health, Laboratory of Medical Statistics, Biometry and Epidemiology “G. A. Maccacaro”, University of Milan, Milan, Italy; 2 Healthcare Professions Department, Fondazione IRCCS Ca’ Granda Ospedale Maggiore Policlinico, Milan, Italy; 3 Cystic Fibrosis Centre, Fondazione IRCCS Ca’ Granda Ospedale Maggiore Policlinico, Milan, Italy; 4 Department of Pathophysiology and Transplantation, University of Milan, Milan, Italy

**Keywords:** Lung clearance index, Cystic fibrosis, Pediatrics, Phenotypes, Time-to-event data

## Abstract

**Background:**

Lung clearance index (LCI) is accepted as an early marker of lung disease in cystic fibrosis (CF), however the utility of LCI to identify subgroups of CF disease in the paediatric age group has never been explored. The aim of the study was to characterize phenotypes of children with CF using LCI as a marker of ventilation inhomogeneity and to investigate whether these phenotypes distinguished patients based on time to pulmonary exacerbation (PE).

**Methods:**

Data were collected on patients with CF aged < 18 years old, attending the CF Center of Milan during outpatient follow-up visits between October 2014 and September 2019. Cluster analysis using agglomerative nesting hierarchical method was performed to generate distinct phenotypes. Time-to-recurrent event analysis investigated association of phenotypes with PE.

**Results:**

We collected 313 multiple breath washout tests on 125 children aged 5.5–16.8 years. Cluster analysis identified two divergent phenotypes in children and adolescents of same age, presenting with almost normal FEV_1_ but with substantial difference in markers of ventilation inhomogeneity (mean LCI difference of 3.4, 95% Confidence Interval [CI] 2.6–4.2). A less severe phenotype was associated with a lower risk of PE relapse (Hazard Ratio 0.45, 95% CI 0.34–0.62).

**Conclusions:**

LCI is useful in clinical practice to characterize distinct phenotypes of children and adolescents with mild/normal FEV_1_. A less severe phenotype translates into a lower risk of PE relapse.

**Supplementary Information:**

The online version contains supplementary material available at 10.1186/s12890-022-01903-5.

## Introduction

Lung clearance index (LCI) is the main derivative of the multiple breath washout test (MBW), and it has gained lot of attention during the last years as a promising outcome measure in cystic fibrosis (CF), complementary to conventional spirometry. Being CF characterized by a progressive lung disease, LCI has denoted itself as an early marker of ventilation inhomogeneity [1], measuring the unevenness of ventilation and thus recognizing early signs of CF lung disease [2], especially in children with a normal/above normal spirometry.

Evidence collected so far has recognized LCI as an outcome measure in interventional trials [3–5]. Only recently, LCI was found sensitive to assess CF disease progression without overt clinical symptoms [6], however the utility of LCI as a clinical marker to guide the multidisciplinary practice of the CF team is still a matter of discussion [7, 8]. Considering that personalized medicine not only aims to target therapies but also to identify individuals at elevated risk that could benefit most from therapies, we hypothesized that LCI can help to characterize clinical phenotypes of children with CF with regards to severity of lung disease.

In order to describe individuals who could benefit most from personalized therapies, we aimed to identify subgroups of children and adolescents with CF using known markers of the disease together with early markers of ventilation inhomogeneity. Furthermore, we investigated whether resulting phenotypes could discriminate young individuals with CF based on time to pulmonary exacerbation (PE).

## Methods

### Study design

With the aim to profile children with CF according to their clinical characteristics, we adopted a cross-sectional design, using data generated on the first MBW assessment to identify clusters (i.e., phenotypes). On the contrary, a 4-year cohort design was considered to explore the association between phenotypes and the risk of PE relapse, conceived as the time since the first MBW performed to date(s) of PE.

The study has been approved by local ethics committee of Fondazione IRCCS Ca’ Granda Ospedale Maggiore Policlinico (456/2021), and written informed consent was obtained from parents or guardians of participants. All research was conducted in accordance with relevant guidelines and regulations.

### Participants

Clinical data were collected on children and adolescents aged < 18 years old, attending the CF Center of Milan during routine outpatient visits between October 2014 and September 2019. As per current clinical practice, children perform MBW test and spirometry as part of their clinical review. For the cross-sectional analysis, only lung function test results from participants in clinical stable condition were considered. Clinical stability was defined as the absence of change in treatment (i.e., antibiotic treatment, systemic corticosteroids), hospital admissions and/or signs and symptoms of pulmonary exacerbation [9]. Further, we included only paediatric individuals with at least two MBW assessments during the study period, in order to have at least one follow-up measure to assess phenotypes over time.

### Pulmonary measures

Lung volumes were measured by a 830 L plethysmograph (Master Screen Body 4.2, E. Jaeger GmbH) in the sitting position, according to ATS/ERS guidelines [10]. Flow and volume were measured by a pneumotachograph with a 0.036 kPa L/-1 s resistance and 160 ml dead volume. Values from spirometry are reported as a percentage of predicted values and as Z score, according to Quanjer’s equation developed under the Global Lung Function initiative [11]. Patients’ lung function was considered in the normal range when FEV_1_ was above the − 1.64 Z score (lower limit of normal at 5th centile).

An open-circuit MBW hard- and software package with nitrogen as tracer gas (MBWN_2_) was used (Exhalyzer® D and Spiroware 3.2.2 Ecomedics AG, CH) and calibration and measurement procedures were performed as suggested [12–15]. Only results from three reproducible runs were considered, defined as a variation of functional residual capacity and LCI (1/40th of the starting concentration) values within 10%. LCI together with convective gas mixing in the conducting airways (Scond*VT) and diffusion–convection interaction within the acinus (Sacin*VT) were therefore recorded, before patients performed spirometry. In children under 6 years old, upper limit of normal (ULN) was calculated based on the equation reported by Lum et al. [16]. For the paediatric age, the cut-off of normality equals to 7.91 was adopted, as recently published [17]. Newly normative data published on LCI Z score were reported as well [17]. An adequate environment with adequate distraction for younger children was assured during each test [14]. Data from earlier software versions were re-run in the version of Spiroware 3.2.2.

### Clinical measures

Along with lung function measures, we collected age, sex, genotype and pancreatic status. Nutritional health was expressed as Z score of body mass index (BMI) based on Italian reference equation [18]. Also, we collected presence of CF-related diabetes (CFRD) and colonization by *Pseudomonas aeruginosa*, which was defined as chronic, intermittent of free [19]. Pulmonary exacerbation was defined as the need for additional antibiotic treatment following change in respiratory signs and symptoms [9, 20]. PEs in the 12 months preceding LCI together with the number of hospitalizations in the previous year due to respiratory symptoms were also used in order to define clusters not only based just on classic features of the disease. For the time-event analysis, we considered PE as an event treated at home or at hospital.

### Statistical analysis

Descriptive statistics were used to summarize demographic and clinical features. Difference between continuous or categorical variables were tested with Student’s t or Wilcoxon rank sum test or Fisher test, according to variables distribution.

The agglomerative nesting (AGNES) hierarchical method using Gower’s distance was the approach selected to generate phenotypes, using variables collected throughout the study, the derivatives of MBWN_2_, namely LCI, Sacin^*VT^ and Scond^*VT^ but not FEV_1_. To determine the optimal number of clusters, elbow and silhouette methods, gap statistics and the visual inspection of dendrogram were taken into consideration. Additional detail on cluster analysis is provided in the Additional file 1.

We assessed the association between phenotypes and subsequent time to PE using the Prentice, Williams and Peterson (PWP) gap time models [21]. PWP models are an extension of Cox regression models; for every subsequent event, the population at risk includes only those with a previous event. The PWP model was used to evaluate if there is an association between phenotype and number of PEs, calculating hazard ratios (HR) of repeated PE relapses. Since 7% of children had more than three follow-up events, risk set groups were not refined further to avoid unreliable estimates. All statistical tests were performed using the open-source software R Core Team, version 3.6.1 [22].

## Results

Between October 2014 and September 2019, 313 MBWN_2_ tests were performed during outpatient clinic visits on 125 children and adolescents with CF, representing the 44.3% (125/282) of eligible CF population at our center.

### Cluster characteristics

Table 1 describes the baseline characteristics of the 125 patients evaluated at least twice, grouped by the two clusters found applying AGNES algorithm. Children who performed only one MBWN_2_ test were excluded (n = 120), since they could not contribute to longitudinal evaluation of phenotypes.

**Table 1:** Sample characteristics stratified by clusters

	Total	Cluster #1	Cluster #2	*P*-value
Subjects, nr	125	78	47	
Age, years	11.4 (2.8)	11.1 (3)	11.8 (2.3)	0.156
Sex, female	61 (48.8)	36 (46.2)	25 (53.2)	0.466
BMI *Z *score	− 0.57 (0.88)	− 0.68 (0.78)	− 0.37 (1.00)	0.073
Pancreatic insufficiency	74 (59.2)	74 (94.9)	–	< 0.001
CFRD	1 (0.8)	1 (1.3)	–	1.000
CFTR genotype				< 0.001
F508del/other	50 (40.0)	32 (41.0)	18 (38.3)	
F508del/F508del	31 (24.8)	30 (38.5)	1 (2.1)	
Other/other	44 (35.2)	16 (20.5)	28 (59.6)	
*Pseudomonas aeruginosa*, free	76 (65)	41 (52.6)	35 (85.4)	< 0.001
FEV_1_% predicted	100.4 (17.4)	95.3 (18.0)	109.0 (12.4)	< 0.001
FEV_1_ Z score	0.05 (1.48)	− 0.38 (1.52)	0.79 (1.08)	< 0.001
FVC % predicted	107.4 (14.7)	104.4 (16.0)	112.4 (10.7)	0.002
FVC Z score	0.61 (1.23)	0.35 (1.34)	1.03 (0.90)	0.001
LCI	10.07 (2.98)	11.33 (2.91)	7.97 (1.57)	< 0.001
LCI Z score, median (IQR)	5.22 (7.89)	8.32 (8.44)	0.98 (2.98)	< 0.001
Sacin^*VT^	0.138 (0.109)	0.161 (0.116)	0.1 (0.084)	0.001
Scond^*VT^	0.066 (0.030)	0.078 (0.024)	0.045 (0.028)	< 0.001
Pulmonary exacerbations^a^, median (IQR)	2 (0–3)	3 (1–4)	0 (0–1)	< 0.001
Hospitalization^a^				< 0.001
1	15 (12.0)	15 (19.2)	–	
⋝2	4 (3.2)	4 (5.1)	–	

Values are expressed as absolute number (percentage) or mean (sd), where not differently expressed

*CFTR* cystic fibrosis transmembrane conductance regulator

^a^Reference time period is 12 months preceding first MBWN_2_ test

**Fig. 1:**
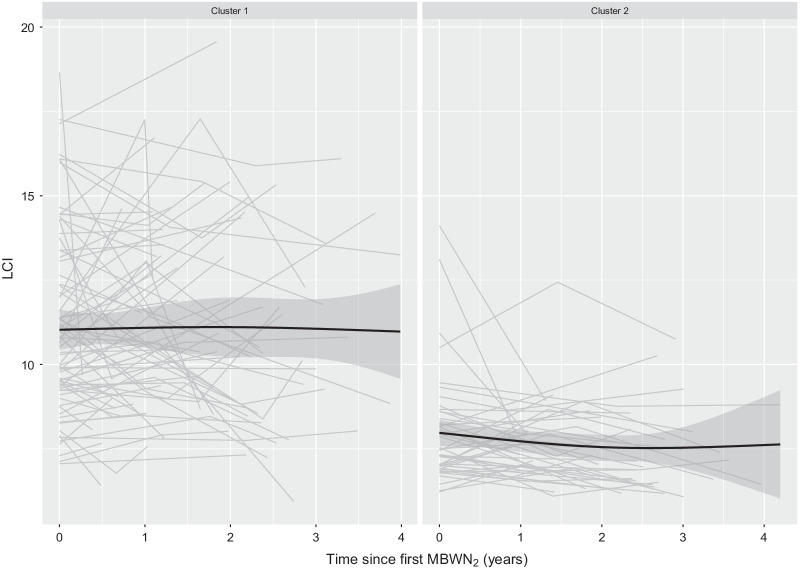
LCI profile for children through-year follow-up visits. Curved line describes the spline function with 3 knots used to visually describe the trajectory of LCI over time. Bands denote 95% CI interval

The average age of participants in the two clusters is very similar. Cluster #1 is characterized by the co-presence of several negative known prognostic factors (i.e., pancreatic insufficiency and presence of *Pseudomonas aeruginosa).* The sole analysis of FEV_1_%predicted returns all patients in the two clusters in the range of moderate to mild/normal air flow obstruction. Particularly, 90.5% and 100% of patients, respectively in clusters #1 and #2, could be considered having mild/normal FEV_1_. These percentages vary if one adopts Z score of FEV_1_, under which case the percentage of patients with a normal lung function drops to 81.1% and to 97.7% in the first and second cluster, respectively. Using MBWN_2,_ ventilation inhomogeneity (i.e., LCI > ULN) characterizes the majority of patients in cluster #1, 92.3% compared to 42.6% in cluster #2. At a population level, 95% Confidence Interval (CI) for the mean LCI in cluster #1 goes from 10.7 to 12.0, whereas 95%CI for the mean LCI in cluster #2 is much lower, namely 7.5–8.4. Acinar and conductive ventilation inhomogeneity is less pronounced in cluster #2 compared to cluster #1. Patients in cluster #1 show also more variability in ventilation inhomogeneity measurements (Fig. 1), whereas patients in cluster #2 look more stable over time. Under these two distinct phenotypic clusters, no sex difference is detectable (*P* = 0.466) nor any significant difference in nutritional status, − 0.34 (95% CI − 0.65 to 0.03) BMI Z score. Age span is pretty much identical between the two partitions, 5.5–16.8 and 7.7–16.3 years, respectively in clusters #1and #2.

### Time to recurrent pulmonary exacerbations in CF phenotypes

Most children had at least one PE (n = 114, 91.2%) during the 4-year follow-up. Mean number of PE was 2.3, varying from 0 to 4. One to 3 relapses of PE occurred in 114, 89, and 28 children, respectively (Table 2).

**Table 2:** Distribution of demographic and clinical markers of CF disease for PE recurrence among 125 children

	Recurrence no		
	First PE (n = 114)	Second PE (n = 89)	Third PE (n = 28)
Age, years	11.3 (2.8)	13.1 (2.9)	14.1 (2.2)
Sex, female	55 (48.2)	41 (46.1)	11 (39.3)
BMI, *Z *score	− 0.59 (0.88)	− 0.62 (0.87)	− 0.83 (0.9)
Pancreatic insufficiency	72 (63.2)	59 (66.3)	23 (82.1)
CFTR genotype			
F508del/other	47 (41.2)	37 (41.6)	11 (39.3)
F508del/F508del	30 (26.3)	27 (30.3)	10 (35.7)
Other/other	37 (32.5)	25 (28.1)	7 (25)
*Pseudomonas aeruginosa*, free	68 (59.6)	50 (56.2)	18 (64.3)
FEV_1_, % predicted	99.4 (17.3)	96.3 (18.1)	93.8 (16.0)
FEV_1_, Z score	− 0.03 (1.46)	− 0.29 (1.52)	− 0.51 (1.35)
LCI	10.3 (2.99)	10.6 (3.44)	10.8 (2.64)
LCI, Z score^a^	5.51 (8.44)	5.64 (8.84)	7.19 (8.64)

Values are presented as mean (standard deviation) or count (percentage)

^a^Median (IQR)

The clinical characteristics became more severe as patients had more events, as summarized by Table 2, although the percentage of children without *Pseudomonas aeruginosa* remained stable over time. According to PWP model, the best phenotype was associated with a lower risk of PE relapse (HR 0.45, 95% CI 0.34–0.62). When estimating the association between phenotypes and PE relapse separately for children having experienced one, two or three follow-up PE events, there was no evidence of difference between the different strata (*P* = 0.102). Overall, the median time with interquartile range to first PE was 0.30 (0.15–0.71) years for children and adolescents in cluster #1 and 1.19 (0.46–2.89) years for patients in cluster #2.

## Discussion

In the present study, derivatives of MBWN_2_ test contributed to characterize phenotypic clusters of children and adolescents with CF in stable conditions. Most interestingly, the association found between relapse of pulmonary exacerbations and phenotypes promotes the importance of LCI when related to the burden of disease, considering how impactful can be pulmonary exacerbations either treated at home or at hospital on children and their families.

Cluster analysis identified two different profiles of patients of the same age, equally represented by boys and girls and without clinically significant difference in nutritional status. Cluster #2 depicts individuals with less severe genotype and better lung status. Interestingly, absence of pancreatic insufficiency uniquely defines individuals in cluster #2. By the contrary, cluster #1 identifies peers with a more severe expression of the disease. The most important aspect to consider is that divergent phenotypes emerged during preadolescence, showing different lung health and a differently compromised lung periphery, only when evaluated by score indices of ventilation inhomogeneity and FEV_1_ Z score together. This is furthermore of interest, taking into consideration that clustering used information derived by MBWN_2_ and not by spirometry. Although statistically different between the two clusters, FEV_1_ remains above 90% predicted. This reinforces its poor sensitivity in discriminating lung health in patients with CF during childhood and adolescence, as already reported elsewhere [2, 23, 24]. The overall compromise of cluster #1 is also supported by the higher prevalence of pulmonary exacerbations compared to cluster #2 and by the absence of hospitalization at all in cluster #2. Practically, children in cluster #1 may be identified at elevated risk, being the target of more personalized interventions. In fact, despite these individuals may present with FEV_1_ in the normal range, their LCI was higher compared to those of same age who were pancreatic sufficient, thus suggesting that tailored and intensive intervention to also treat lung periphery is appropriate. As commented by Nyilas et al., children with different phenotypes and particularly with distinct ventilation inhomogeneity profiles, may benefit from different therapeutic approaches, such as aerosol therapy performed with distinct devices in order to deliver different particle sizes [25] as well as physiotherapy interventions targeting small or large airways, or different scheduling of follow-up visit. A few cluster-based approaches are reported in the literature about CF [26–29], and it is worth to be mentioned that adolescence was the time when individuals with rapid FEV_1_ decline differentiate from other phenotypes characterized by slower lung function decline [26]. Our findings showed divergent phenotypic characteristics already at 11 years old, thus underlying the importance of introducing early markers of lung functional status along the routine CF care. So far, only one study used LCI to define physiological phenotypes in children with different lung diseases, including CF [25], showing how determinant was using MBW test to characterize children with normal and abnormal MBW derivatives.

In the present study, physicians and healthcare staff were not blinded to MBW assessment, therefore it is questionable if the steady trend in LCI observed in our study, especially in children defined by cluster #2, should be attributed to any medical decision and subsequent intervention triggered by the evaluation of LCI during the routine follow-up visits. In a recent study [30], an increase in LCI larger than 1 unit was not associated with increased antimicrobial use or pathogens load over 2-year follow-up based on LCI-triggered bronchoalveolar lavage. However, an increase in LCI over time between 0.24 and 0.29 units/year was just reported [6], suggesting that even small fluctuations may be required to trigger change in the multidisciplinary management of CF, especially in children and adolescents with stable conditions. Thus, it is plausible that such small variations could be currently used in a clinical setting to guide therapies or to require additional assessment. Indeed, it is to be recalled how meaningful was the rescue of MBW test from the armoury of lung function tests in the 2010s CF scenario worldwide. It would not be surprising if higher LCI values than 7—which was considered a standard cut-off of normality back in time—could have opened to clinicians more options in the care of their patients. It is well known that information with a high emotional impact can alter the decisional process, even though the probabilistic rules to guide decisions are already there. Anyway, the observed stability in ventilation inhomogeneity is encouraging for our CF team, especially considering the recent findings from Sandvik et al. [31], showing that no progression of structural lung disease at CT scan is expected in children with stable LCI.

Our findings describe for the first time an association between phenotypes based on ventilation inhomogeneity and the risk of PE relapse in children and adolescents with CF. The conditional model of PWP with gap time particularly fits the data on PEs in CF, since it does not allow to consider an individual at risk until the end of the previous episode.

Earlier, Vermeulen et al. [32] showed an association between baseline LCI and FEV_1_ and time to first PE in a cohort of 5–19 years old patients with CF. Annual PE rate was higher in children with the worst Z score of LCI and FEV_1_, however LCI Z score was identified as the only predictor of the PE rate in the 12 months following the baseline assessment. Their methods relied on Kaplan–Meier and Negative Binomial regression, which assume that each patient has recurrent events according to individual Poisson event rate which in turn varies according to a specific distribution across patients. Negative Binomial regression model seems appropriate to estimate recurrent events when information on time is not available [33], differently from the present study, in which we collected the exact time of PE throughout the follow-up. Moreover, the study from Vermeulen et al. used information on PEs up to first event only, potentially leading to an inaccurate evaluation of the association of selected covariates with an event that occurs more than once and that are possibly dependent. However, both these studies show how useful was MBWN_2_ to capture relevant association with the course of CF disease. Furthermore, these results remind us of the paramount importance to delay the occurrence of PE and to concentrate the efforts of the multidisciplinary team to preserve lung function, especially in terms of ventilation homogeneity in children.

### Strength and limitations

Cluster analysis is a multivariate method aimed to classify individuals based on a set of measured variables. By consequence, the variable selection is a crucial step and could have been done differently from the one we are proposing here, which relied on a minimum set of widely available CF clinical characteristics. With regard to phenotypic clusters, further steps should take into consideration the anatomical correlates of CF lung disease to address to each cluster a specific level of anatomical damage. Despite these limitations, our study is characterized by several unique strengths. Not only we generated clusters based on a large cohort of Italian children and adolescents with CF using LCI but also, we adopted an *ad-hoc* statistical approach to evaluate the association between phenotypes and PE relapses, which are considered high-impactful events in patients and their families’ life, therefore assuming a unique relevance under the patient’s perspective.

## Conclusion

LCI is useful in the clinic to characterize ventilation inhomogeneity in distinct phenotypes of children and adolescents with mild/normal FEV_1_. A less severe phenotype translates into a lower risk of pulmonary exacerbation relapse. Altogether these findings add to the available literature in confirming the clinical utility of MBWN_2_ to monitor lung disease during childhood and adolescence in CF.

## Supplementary Information

**Additional file 1: Table S1.** Validity and stability indices and other descriptive clusters properties.

## Data Availability

The datasets generated during and analyzed during the current study are not publicly available due to privacy restrictions but are available from the corresponding author on reasonable request.

## Abbreviations

LCI: Lung clearance index
MBW: Multiple breath washout
CF: Cystic fibrosis
PE: Pulmonary exacerbation
FEV_1_: Forced expiratory volume in the first second
MBWN_2_: Nitrogen multiple breath washout
ULN: Upper limit of normal
BMI: Body mass index
CFRD: CF-related diabetes
AGNES: Agglomerative nesting
PWP: Prentice, Williams and Peterson
IQR: Interquartile range
CI: Confidence interval
