# Supplementary material for: Lung clearance index to characterize clinical phenotypes of children and adolescents with cystic fibrosis
Source: BMC Pulm Med. 2022 Apr 1;22:122. doi: 10.1186/s12890-022-01903-5 (PMC8976307; doi:10.1186/s12890-022-01903-5)
Supplement: Supplementary file 1 — Additional file 1: Table S1. Validity and stability indices and other descriptive clusters properties. [file 12890_2022_1903_MOESM1_ESM.pdf]

Additional File 1

Lung Clearance Index to characterize clinical phenotypes of children and adolescents with cystic fibrosis

Simone Gambazza, Federico Ambrogi, Federica Carta, Laura Moroni, Maria Russo, Anna Brivio, and Carla Colombo

METHODS

Clustering was the approach selected to generate phenotypes based on the variables collected throughout the study. In adjunct to age, sex and *CFTR* genotype, clustering was based also on nutritional status, i.e., BMI Z-score and pancreatic exocrine function, on the presence of CFRD, and on colonization by *Pseudomonas aeruginosa.* To characterize lung disease, indices derived by $MBWN_2$ test were used, namely LCI, $Sacin^{*VT}$ and $Scond^{*VT}$. The number of pulmonary exacerbations in the twelve months preceding $MBWN_2$ together with the number of hospitalizations in the previous year were also used in order to define clusters not only based just on classic markers of the disease.

The use of Gower's distance limited the possible algorithms to be explored in order to identify clusters within this CF cohort, and therefore the final choice was made between two *bottom-up* hierarchical methods, i.e. AGglomerative NESting (AGNES) and Hierarchical CLUSTering (hclust), and one partitioning method, i.e. Partitioning Around Medoids (PAM), an approach which is based on medoids, a robust alternative to k-means. For hierarchical methods, the best linkage function was selected comparing correlation coefficients between cophenetic and the original distance, resulting from different clustering methods: average, single, complete and Ward.

Silhouette coefficient and Dunn index were used to describe the goodness of clustering, whereas consistency measures were obtained by adopting the *clValid* package (1), available within R. The number of clusters was two according to the majority of indices evaluated (Table 1).

Table S1. Validity and stability indices and other descriptive clusters properties

	AGNES (k=2)	PAM (k=2)	Hclust (k=2)	*Reference*
Linkage	Ward	-	Average	
Metric	Gower	Gower	Gower	
Correlation coefficient	0.59	-	0.60	*>0.80*
Agglomerative coefficient	0.96	-	-	
Silhouette Index	0.33	0.31	0.35	*>0.50*
Dunn Index	0.20	0.10	0.27	*Highest*
Average within	0.22	0.25	0.29	*Lowest*
Average between	0.35	0.35	0.44	*Largest*
APN	0.0961	0.0113	0.0961	*Lowest*
AD	15.115	12.415	15.115	*Lowest*
ADM	0.1501	0.0168	0.1501	*Lowest*
FOM	0.0987	0.0840	0.0987	*Lowest*

APN = Average proportion of non-overlap; AD = Average Distance; ADM = Average distance between means; FOM = Figure of merit.

Despite the best performance among all the indices shown by *hclust*, the analysis of dendrogram revealed a severe imbalance in the number of subjects within each cluster, 2 patients in the first and 123 in the second cluster, therefore this approach was discarded as clinically useless. AGNES showed a slightly better performance in the validity of its clustering structure against poorer stability, compared to PAM.

REFERENCE

1. Brook G, Pihur V, Datta S, Datta S. clValid: An R Package for Cluster Validation. J Stat Softw. 2008;25(4).
